# MRSA at patient admission: the right question to identify the colonized patient

**DOI:** 10.1186/2047-2994-4-S1-P190

**Published:** 2015-06-16

**Authors:** A Bispo, C Palos

**Affiliations:** 1 Infection Control and Antibiotics Committee, Hospital Beatriz Ângelo, Loures, Portugal

## Introduction

Portugal has one of the highest MRSA infection and colonization rates in Europe, so it becomes critical to identify MRSA colonized patients at hospital admission on the Emergency Room (ER), before placement into wards, preventing the mixing of colonized patients with non-colonized ones. Infection Control and Antibiotics Committee (ICAC) developed an Electronic Epidemiological Query at Admission (EEQA) to be answered at patient admission. One of the questions targets MRSA colonization risk.

## Objectives

The objective of our study was to identify the positive predictive value (PPV) of the question that targets the MRSA high risk patient:***Hospitalization or long term care admission for at least 3 days in the last 3 months, or tracheostomy?***

## Methods

With the approval of the Clinical Board, the ICAC defined as mandatorily the fulfillment of EEQA to all patients at admission. A positive answer to this question triggers automatically (and without the doctors approval or intent) nasal and perianal screening swabs for MRSA. Infection Control nurse activates pre-emptive contact isolation precautions immediately. Swab results are known after 48h and then precautions can be stopped or room isolation is implemented.

## Results

In 2014 we had 13.893 EEQA submitted; from these, 3.523 (23,36%) were positive for at least one of the 8 questions. The question that targets MRSA colonized risk patient had a positive answer in 3.082 EEQA (87,4% of the positive EEQA, and 22,1% of all EEQA submitted). A PPV value of 24,6%, with 759 cultural positive screening results in 3.082 identified risk patient.

## Conclusion

Using the EEQA, we’ve obtained a PPV of 24,6 % for the question choosed to identify and screen colonized MRSA risk patients. This value represents 759 MRSA patient admissions in one year, who received adequate isolation procedures, preventing their mixing with non MRSA colonized patients during hospital stay. This may had a decisive role on observed reduction of MRSA nosocomial infection rate at our hospital.

## Disclosure of interest

None declared.

